# Intramural duodenal hematoma secondary to pancreatitis: case report and review of the literature

**DOI:** 10.1590/1516-3180.2017.0134290517

**Published:** 2017-12-07

**Authors:** João Henrique Botto de Oliveira, Raiza Samenica Esper, Rodrigo Campos Ocariz, Flora Specian Sartori, Lucas Marcelo Dias Freire, Elinton Adami Chaim, Francisco Callejas-Neto, Everton Cazzo

**Affiliations:** I MD. Resident Physician, Department of Surgery, Centro Médico de Campinas (CMC), Campinas (SP), Brazil; II MD. Attending Physician, Endovascular Surgery Unit, Centro Médico de Campinas (CMC), Campinas (SP), Brazil; III MD, PhD. Full Professor, Department of Surgery, Faculdade de Ciências Médicas da Universidade Estadual de Campinas (FCM/UNICAMP), Campinas (SP), Brazil.; IV MD, MSc. Assistant Professor, Department of Surgery, Faculdade de Ciências Médicas da Universidade Estadual de Campinas (FCM/UNICAMP), Campinas (SP), Brazil.; V MD, PhD. Adjunct Professor, Department of Surgery, Faculdade de Ciências Médicas da Universidade Estadual de Campinas (FCM/UNICAMP), Campinas (SP), Brazil.

**Keywords:** Pancreatitis, Duodenum, Hematoma, Embolization, therapeutic, Duodenal diseases

## Abstract

**CONTEXT:**

Spontaneous intramural duodenal hematoma is uncommon and is usually associated with coagulopathy, anticoagulant therapy and endoscopic procedures. The aim here was to describe a case of intramural duodenal hematoma caused by chronic exacerbation of pancreatitis.

**CASE REPORT:**

A 46-year-old male with chronic alcoholic pancreatitis was admitted to hospital due to abdominal pain, melena and low hemoglobin. An intramural duodenal hematoma with active bleeding was detected and selective angioembolization was warranted. The patient evolved with a perforated duodenum and underwent laparotomy with exclusion of the pylorus and Roux-en-Y gastrojejunostomy. He was discharged nine days later.

**CONCLUSION:**

Intramural duodenal hematoma is a rare complication of pancreatitis. Selective embolization is the preferred treatment for hemorrhagic complications of pancreatitis. However, the risk of visceral ischemia and perforation should be considered.

## INTRODUCTION

The first description of an intramural duodenal hematoma was made by McLauchlan in 1838. This condition is usually associated with blunt abdominal trauma. Spontaneous intramural duodenal hematoma is uncommon and has been linked to coagulopathy, anticoagulant therapy and endoscopic procedures.^1^^,^^2^^,^^3^ Other causes include several pancreatic diseases, collagenosis, peptic ulcers and pancreaticoduodenal aneurysm.^4^^,^^5^^,^^6^^,^^7^ To date, the exact mechanism leading to intramural hematoma in cases of pancreatitis has not yet been fully elucidated and the prognosis has not yet been completely defined, mainly due to its scarcity.^1^^,^^3^^,^^5^^,^^6^^,^^7^^,^^8^^,^^9^

This study sought to describe a case of an intramural duodenal hematoma caused by chronic exacerbation of pancreatitis.

## CASE REPORT

The patient (JCAP) was a 43-year-old white male who had been a chronic abuser of alcohol for 25 years (two liters of distilled liquor/day), with an antecedent of acute pancreatitis five years before the present case report. He had been complaining of strong typical pain for three days, along with melena.

**Figure 1:**
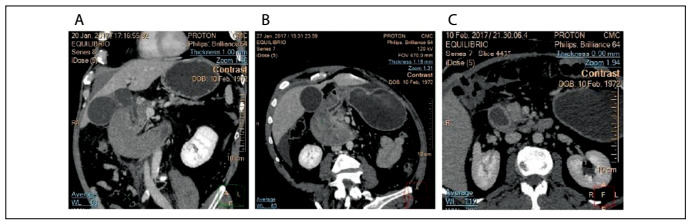
Computed tomography scans: A) at admission; B) post-embolization; C) post-surgical control.

**Figure 2:**
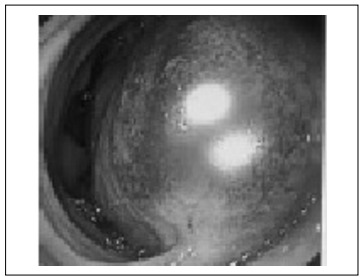
Upper gastrointestinal endoscopy.

At admission to hospital, the following test results were noted: leukogram = 15,000 u/l, hemoglobin = 16 g/dl and amylase = 99 IU/l. A computed tomography (CT) scan showed signs of chronic pancreatitis and a bulky submucosal duodenal hematoma from the bulb to the third portion of the duodenum with intramural active bleeding in the region of the gastroduodenal artery (Figure 1A). Upper digestive tract endoscopy revealed a large submucosal hematoma in the duodenum, without any active bleeding into the lumen (Figure 2).

**Figure 3:**
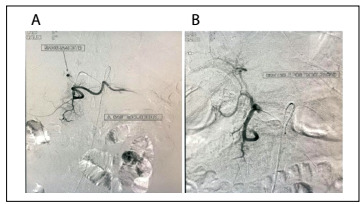
Arterial embolization: A) active bleeding in the region of the gastroduodenal artery; B) post-embolization control.

On the following day, the hemoglobin level decreased to 12 g/dl and selective angioembolization was indicated. During angiography, active bleeding was detected (Figure 3A). After embolization, no more signs of active bleeding were observed (Figure 3B).

One day after this procedure, there was worsening of the pain. A CT scan showed signs of visceral perforation (Figure 1B). Emergency laparotomy was warranted and revealed the following: an ischemic duodenum with a bulky wall hematoma and a 6-cm duodenal rupture from the bulb to the second portion of the duodenum, along with signs of acute pancreatitis. The duodenal rupture was closed, along with exclusion of the pylorus, and Roux-en-Y gastroenteroanastomosis was carried out.

The patient then progressed with a high-output duodenal fistula. Treatment consisting of parenteral nutrition, octreotide and antibiotic therapy was started, and this led to regression over a nine-day period. A control CT scan demonstrated regression of the hematoma, while the signs of chronic pancreatitis continued to be present, but without evidence of exacerbation (Figure 1C). A contrasted upper gastrointestinal radiographic series showed exclusion of the pylorus and patent gastroenteroanastomosis. The patient was then followed up for six months, with uneventful evolution.

## DISCUSSION

Duodenal hematomas have been described as complications of both acute and chronic pancreatitis. Acute pancreatitis is a common disease, with an incidence of 20 to 40 cases/100,000 person-years of life and a mortality rate close to 5%, and the vast majority of the cases are of biliary etiology.^10^ On the other hand, chronic pancreatitis is a progressive inflammatory disorder characterized by irreversible destruction of the pancreatic parenchyma and may be associated with disabling chronic pain and permanent loss of exocrine and endocrine function. The majority of such cases are alcohol-related. Their prevalence is 3-4 cases/100,000 people.^11^

One of the rarest and most fatal complications of pancreatitis is spontaneous bleeding of intestinal vessels. A review of the literature was conducted through an online search for the MeSH terms “duodenum”, “hematoma” and “pancreatitis” in MEDLINE (via PubMed) and LILACS (via BVS) in papers published over the last 20 years (Table 1). We included original studies that reported single cases or case series of this disease. All the papers were checked according to their titles and abstracts (screening). Full papers were obtained from journals available through the website of the Commission for Improvement of Higher Education Personnel (Comissão de Aperfeiçoamento de Pessoal de Nível Superior, CAPES), of the Brazilian Ministry of Education. Articles that were unavailable were requested from their authors. Articles presenting potentially relevant studies were read and analyzed to assess the inclusion criteria. We excluded articles that consisted of *in vitro* or animal studies, articles in which the participants’ characteristics did not match those mentioned above, poster session abstracts, review articles and other types of publications. Articles that described traumatic or iatrogenic duodenal hematomas were also excluded. Other papers were used for contextualization and discussion.

**Table 1:** Database search results for duodenal hematomas caused by pancreatitis

Electronic databases	Search strategies	Results
MEDLINE (PubMed)	(Duodenum) AND	13 case reports
	(Hematoma) AND	
	(Pancreatitis)	
LILACS (BVS)	(((Duodenum) OR	1 case report
	(Duodeno)) AND	
	(Hematoma) AND	
	((Pancreatitis) OR	
	(Pancreatite) OR	
	(Pancreatítis)))	

**Figure 4:**
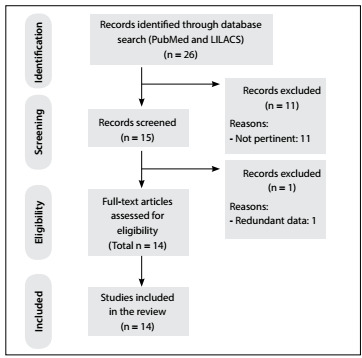
Flow diagram of the narrative review of the literature.

After extensive online research, we identified 14 studies, which were all single case reports. Table 2 ^1^^,^^2^^,^^3^^,^^4^^,^^5^^,^^6^^,^^7^^,^^8^^,^^9^^,^^12^^,^^13^^,^^14^^,^^15^^,^^16^ summarizes the main articles found and their reported outcomes. Figure 4 presents a flow diagram of the articles selected.

**Table 2:** Previously reported cases of intramural duodenal hematoma secondary to pancreatitis

Author	Age (years)	Gender	Etiology of pancreatitis	Anticoagulant/antiplatelet therapy or coagulopathy	Treatment	Outcome
Bodnár et al.^1^	33	Male	Hypertriglyceridemia	No	Conservative	Evolved with a pancreatic abscess that required CT-guided aspiration; no specific therapy for the duodenal hematoma; discharged after six weeks
Leundji et al.^2^	45	Male	Alcoholic	Yes (thrombopenic due to portal hypertension)	Conservative	Asymptomatic one year afterwards
Eurboonyanun et al.^3^	27	Male	Alcoholic	No	Conservative	Uneventful; discharged after 17 days
Dugernier et al.^4^	32	Male	Biliary	No	Conservative	Evolved with infected necrosis that required repeated surgical debridement and drainage; no specific therapy for the duodenal hematoma was carried out due to the poor clinical condition; discharged after six months
Fukunaga et al.^5^	49	Male	Alcoholic	No	Surgical drainage and biopsy	Uneventful; discharged after 18 days
Neuzillet et al.^6^	62	Male	Alcoholic	No	Pancreaticoduodenectomy	Uneventful
Lee et al.^7^	47	Male	Dengue fever	Yes (dengue hemorrhagic fever)	Conservative	Evolved with a peripancreatic abscess that required drainage 51 days after admission; uneventful evolution after drainage
Lee et al.^8^	55	Male	Alcoholic	No	Endoscopic drainage	Uneventful after drainage; follow-up CT demonstrated a smaller intramural mass in the duodenum and upper endoscopy showed a small duodenal ulcer
Veloso et al.^9^	64	Male	Unknown	Yes (aspirin and clopidogrel due to a previous myocardial infarction)	Conservative	Uneventful; discharged after 14 days
Ma et al.^12^	32	Male	Alcoholic	No	Pancreaticoduodenectomy after failure of conservative therapy	Discharged two weeks after surgery; uneventful late postoperative evolution
Dubois et al.^13^	55	Male	Alcoholic	No	Conservative	Uneventful
Khurana et al.^14^	73	Female	Pancreatic malignancy	Yes (warfarin for deep venous thrombosis)	Conservative	Uneventful; after resolution of duodenal hematoma, the patient underwent distal pancreatic resection
Farhoud et al.^15^	71	Female	Obstructive	Yes (warfarin for deep venous thrombosis)	Conservative	Uneventful; discharged after 10 days
Fesenmeyer et al.^16^	71	Male	Unknown	No	Conservative	Uneventful

CT = computed tomography.

Vascular erosion occurs due to extravasation of proteolytic enzymes. Potentially fatal bleeding, characterized by a decrease of two hemoglobin points, are rare complications occurring in about 1% to 5% of patients with acute or chronic pancreatitis. The vessels most affected are the gastroduodenal, pancreatoduodenal, splenic, gastroepiploic and left gastric arteries, because of their proximity to the pancreas, along with small branches of the inferior mesenteric arteries. The symptoms include pain, melena, hematemesis and retroperitoneal bleeding. Upper endoscopy, CT scans and angiography are involved in making the diagnosis.^17^

The majority of the previously reported cases showed that conservative therapy was possible, but this method is associated with lengthier hospital stay and a need for transfusion of blood components. Interventional therapies may include surgical repair using endoscopic and endovascular techniques. The benefits of endoscopy include its minimally invasive nature, compared with other treatment options, and the absence of radiation exposure. However, it has limitations, given that it cannot be used for areas of bleeding that are inaccessible to the intestinal lumen.^8^ Arterial embolization seems to be effective for management of bleeding. Embolization is considered successful when both radiologically and clinically there is evidence of bleeding control characterized by hemoglobin stabilization and absence of signs and symptoms of shock.^18^^,^^19^ Laparotomy for bleeding therapy should only be considered for hemodynamically unstable patients. Advances in endovascular radiology have led to this method becoming the preferred treatment option.^18^

The present case demonstrates the need for a high degree of suspicion that the diagnosis could comprise bleeding caused by pancreatitis, as well as the need to remain open to the possibility of a severe and rare complication after embolization (visceral perforation). This complication requires rapid intervention, because the risk of mortality that it presents is up to 10 times greater than that commonly observed in cases of acute pancreatitis.^20^^,^^21^ The currently available evidence consists solely of single case reports, which thus precludes final conclusions regarding the optimal therapy. Hence, further research is necessary.

## CONCLUSION

Intramural duodenal hematoma is a rare complication of pancreatitis. Selective embolization is the preferred treatment, but the risk of visceral ischemia and perforation should be considered.
